# Effect of SO_2_ Dry Deposition on Porous Dolomitic Limestones

**DOI:** 10.3390/ma3010216

**Published:** 2010-01-07

**Authors:** Mihaela Olaru, Magdalena Aflori, Bogdana Simionescu, Florica Doroftei, Lacramioara Stratulat

**Affiliations:** 1 Petru Poni Institute of Macromolecular Chemistry, 41 A Gr. Ghica Voda Alley, 700487, Iasi, Romania; 2 University of Oviedo, Department of Geology, Calle Jesus Arias de Velasco s/n, 33005 Oviedo, Spain; 3 Moldova National Museum Complex, 1 Stefan cel Mare si Sfant Square, 700028, Iasi, Romania

**Keywords:** limestone, silsesquioxane, nanocomposite

## Abstract

The present study is concerned with the assessment of the relative resistance of a monumental dolomitic limestone (Laspra – Spain) used as building material in stone monuments and submitted to artificial ageing by SO_2_ dry deposition in the presence of humidity. To investigate the protection efficiency of different polymeric coatings, three commercially available siloxane-based oligomers (Lotexan-N, Silres BS 290 and Tegosivin HL 100) and a newly synthesized hybrid nanocomposite with silsesquioxane units (TMSPMA) were used. A comparative assessment of the data obtained in this study underlines that a better limestone protection was obtained when treated with the hybrid nanocomposite with silsesquioxane units.

## 1. Introduction

One of the most aggressive atmospheric pollutant affecting the building materials is sulphur dioxide (SO_2_) which is very reactive and corrosive and acidifies the rain (the so-called “acid rain”). Acid deposition is a more precise acid rain term, which has two main parts: dry and wet. Dry deposition refers to the deposition of pollutant gases and particles in the absence of rain. Wet deposition is concerned with the incorporation of pollutant substances in cloud droplets (occult deposition or rain-out), or in regular precipitation (wet-only precipitation, acid rain or wash-out). Dry deposition, which is more important than wet deposition for highly polluted areas [1], results from the transfer of pollutant gases and/or particles, including aerosols, from the atmosphere to a surface in the absence of rain (short range deposition). The main effects of sulfur dioxide on limestones are the formation of crusts and the loss of material due to solubilization, which can represent the 30–50% of material loss [2]. The loss of material can also be produced where weathering crusts reach certain thicknesses and then drop from the stone surface [3]. The stone surface where the crust is detached usually presents disaggregation and higher porosity and surface area than the original stone, becoming weaker to further weathering processes [4].

A commonly used measure to protect limestones is the application of coatings which isolate them from aggressive environmental factors. Such coatings, particularly when applied upon rain-protected surfaces, have often been found more harmful than if the limestone was left untreated. The reasons for the accelerated decay include the entrapment of water and water vapor in stone whereby the rate of reaction is enhanced. Further, many coatings absorb SO_2_, which also accelerates the reaction rate. Water protective materials such as acrylic copolymers and siloxanes are widely used for the formulation of protective coatings on cultural heritage monuments due to their good adhesion and film forming properties, low viscosity and deep penetration within the stone, ability to crosslink *in situ* after the treatment, as well as to their good weather resistance [5,6,7]. They have environmental stability [8] and have been largely used in conservation practice as coatings, consolidants and adhesives. These materials act as surface modifiers altering the physico-structural properties of the porous materials and changing the physico-chemical behavior of the interface between the work of art and the environment [9]. So, the characterization of the solid surfaces before and after the application of these materials is important for the evaluation of their ability to protect the historic monuments and buildings.

The present study’s aim is the development of a silsesquioxane-based nanocomposite coating presenting improved properties against SO_2_ action as compared to the existing commercial products for the preservation of cultural stone monuments [10,11,12]. Silsesquioxane-based hybrid composites are used as consolidants [13] or water repellents [14] for stone conservation. The selected commercial products are generally applied as water repellent protective treatments for natural stones, offering sometimes protection against pollution, as well. As follows, the protection capacity of the studied products against SO_2_ action on the untreated and treated limestone samples, (Laspra – typical for the region of Asturias, Spain) was assessed. In this context, the stone was coated with three commercially available siloxane-based chemical products and with the newly synthesized hybrid nanocomposite with silsesquioxane units, then submitted to SO_2_ atmosphere in the presence of humidity for 21 days, the protectiveness of the treatments being evaluated through scanning electron microscopy (ESEM), Fourier Transform Infrared Spectroscopy (FTIR) and X-ray analysis, as well as water vapor permeability and color measurements.

## 2. Results and Discussion

Gypsum is produced by reaction between sulphur compounds (SO_2_, SO_3_, H_2_SO_4_), water (liquid or vapor) in the atmosphere and calcite contained in stones and is more soluble and occupies more volume that the original matrix, calcium carbonate. In case of dry deposition, the formation of gypsum from the reaction of calcium carbonate with sulfur dioxide (Equation 1) can be schematized as:

CaCO_3_ (SO_2_/ H_2_O) → CaSO_3_∙1/2 H_2_O CaSO_3_∙1/2 H_2_O (O_2_/H_2_O) → CaSO_4_∙2H_2_O (1)

Another possibility is the absorption of sulfur dioxide in rainwater, liquid atmospheric aerosols, or moist film supported on a stone surface (wet deposition), where it is oxidized to form a sulfuric acid solution that dissolves the calcium carbonate by gypsum formation:

SO_2_ (O_2_/H_2_O) → H_2_SO_4_

CaCO_3_ + H_2_SO_4_ → H_2_O + CaSO_4_∙2H_2_O

With respect to dolomite stones, the basic reaction is likely to imply the formation of gypsum and epsomite (Equation 2):

CaMg(CO_3_)_2_ + 9H_2_O + 2SO_2_ + O_2_ → CaSO_4_∙2H_2_O + MgSO_4_∙7H_2_O + 2CO_2_ (2)

A microscopic investigation was performed in order to assess limestone’s surface morphology. The experiments have shown differences in the habit of the gypsum crystals formed resulted as a consequence of the specific water repellent compound applied onto the stone surface. The gypsum crystals produced during the SO_2_ deposition experiments on stones can occur basically in three form types: (i) prismatic, (ii) sheetlike (or as rosettes when crystals are twinned) and (iii) needlelike [9]. With reference to stone buildings, crusts comprising sheetlike or rosette crystals correspond to advanced stages of deterioration, while needlelike crystals represent the first stages of weathering.

**Figure 1:**
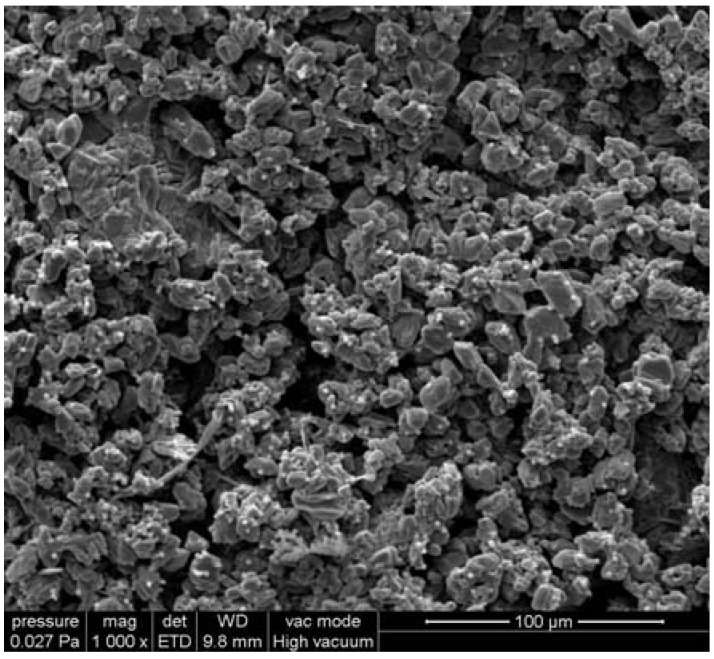
ESEM micrograph of Laspra after exposure to SO_2_ contaminated atmosphere.

For the untreated Laspra samples subjected to SO_2_ action in the presence of humidity, ESEM micrographs evidence the presence of gypsum crystals with a prismatic and bladed appearance, suggesting an accelerated degradation and mass loss (Figure 1).

**Figure 2:**
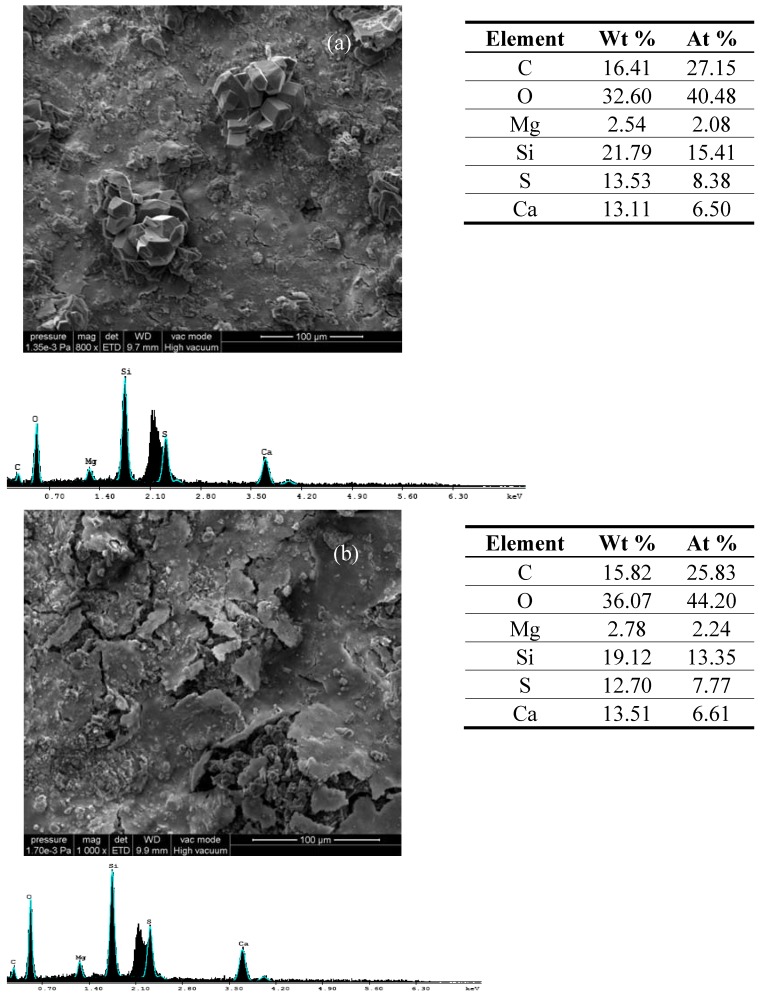

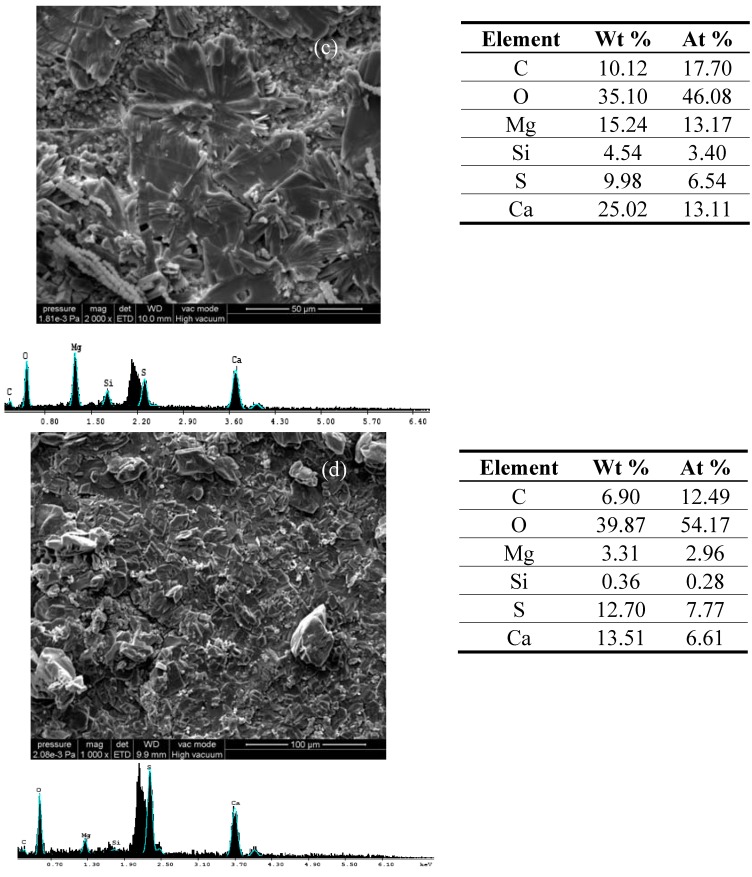
ESEM micrographs, EDX diagrams and elemental composition of Laspra coated with (a) Lotexan-N, (b) Silres® BS 290, (c) Tegosivin HL 100, (d) TMSPMA, after exposure to SO_2_ contaminated atmosphere.

In case of treated stones exposed to SO_2_ action, ESEM images reveal the presence of predominantly gypsum crystals and their sub-efflorescence onto limestones surfaces (a, b, c), as well as the formation of rosettes (d) (Figure 2). Reddy and Leith [15] developed a predictive model for sulphur transport into limestone and marble based on unsteady diffusion into a semi-infinite slab to describe the temporal and spatial surface distribution of sulfate in these stones. This diffusion assumes that a saturated gypsum solution is present within the pores situated on stone surface and that sulfate is transported into the stone’s interior by molecular diffusion. This model could be applied to explain the deterioration mechanism for all studied samples.

**Figure 3:**
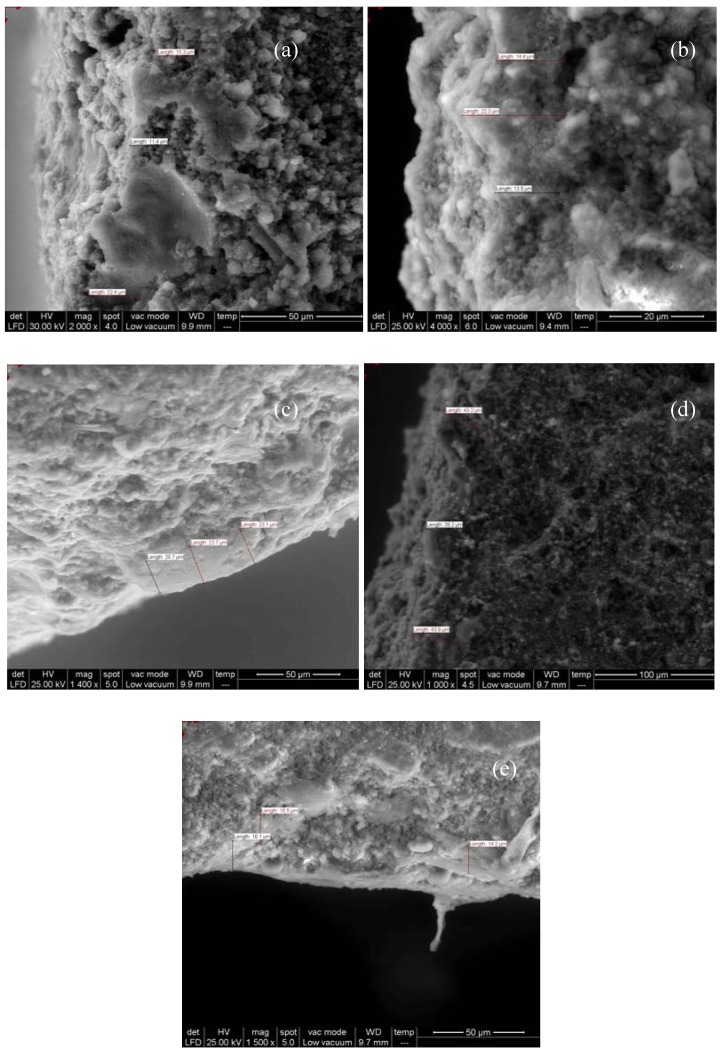
Cross-section SEM images of untreated (a) and treated Laspra with (b) Lotexan-N, (c) Silres® BS 290, (d) Tegosivin HL 100, (e) TMSPMA, after exposure to SO_2_ atmosphere.

**Figure 4:**
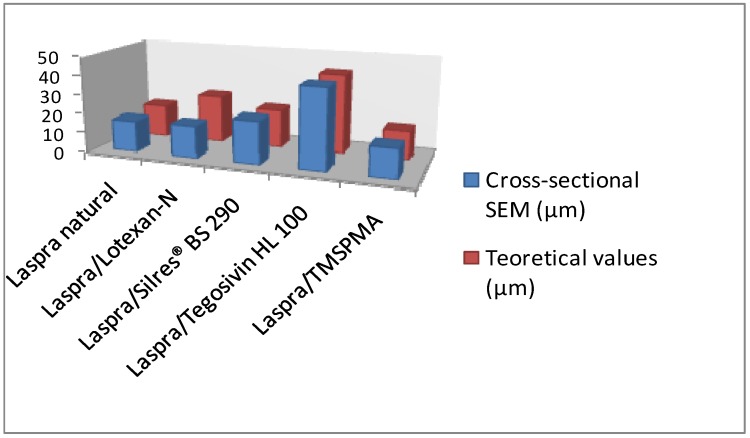
Comparison of gypsum crust thickness obtained through cross-section SEM images and theoretical calculation.

Both theoretical calculation and cross-sectional SEM images were taken into account in order to evaluate the thickness of the gypsum crust thickness (Figure 3, Figure 4). Due to the large molecular structures of the commercial products, they allow a good SO_2_ penetration into the stone substrates, the higher penetration, the higher in-depth sulphation (reaction between carbonate and SO_2_). The higher in-depth sulphation (reaction between carbonate and SO_2_) is sustained by the higher crust thickness after the exposure to SO_2_ atmosphere (Figure 3).

The cross-section SEM images and theoretical calculation revealed similar values for gypsum crust thickness (Figure 4), as follows.

The cross-sections of the limestone coated with the commercial products showed the formation of a homogeneous and compact layer with a thikness of 16.2 µm (Lotexan-N), 13.9 µm (Silres® BS 290) and 9.6 µm (Tegosivin HL 100). Taking into account the thickness of the gypsum crust, as well as the coating layer one, a higher in-depth sulphation can be estimated in the following order: Lotexan-N > Silres® BS 290 > Tegosivin HL 100.

In case of the compound TMSPMA, the nanosize porous network inhibits SO_2_ penetration (M_SO2_ = 64, M_H2O_ = 18), but allows water vapor to enter and exit, letting the limestone “breathe” freely. Therefore, due to the presence of homogeneous layers of TMSPMA nanocomposite, the sulphation proceeds only above the surface, leading to the development of a thin gypsum crust. Moreover, ESEM investigation shows the presence of only sheetlike gypsum crystals. Taking into account that all samples have been exposed under the same conditions, the formation of a thin crust of rosette type (which corresponds to the final stage of weathering) may indicate that the sulphation process in this particular case is stopped at this level, while the appearance of needlelike crystals (initial stage) in case of the commercial product can be connected with the extension of the sulphation process by the formation of subsequent new layers of sulphite and sulphate due to the presence of the water delivered by epsomite or by the one „entrapped” inside the stone and delivered to the surface through the fractures. Moreover, due to trimethoxysilane groups, TMSPMA self-assembles across the surface and bonds with the stone. The solvent (ethanol) evaporates over the curing period, leaving a uniform thin layer of 3.6 µm over the surface. TMSPMA nanocomposite does not form only as a thin coating on the surface, but penetrates the limestone deep inside (aprox. 0.8 cm), deeper than the commercial products. For this reason, this coating could extend the limestone resistance to SO_2_ action.

**Figure 5:**
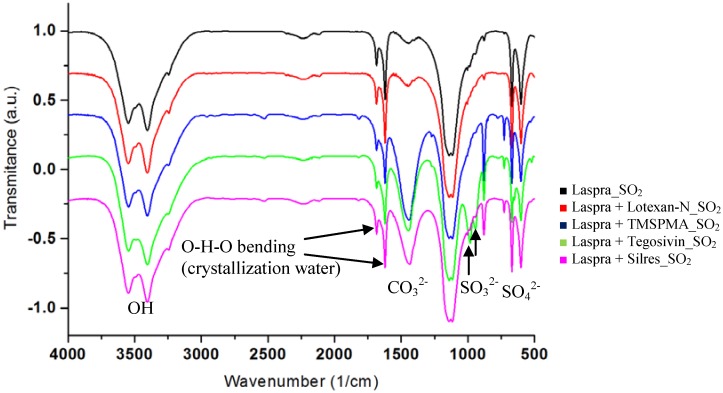
FT-IR spectra of untreated and treated Laspra – subjected to SO_2_ action.

The FTIR spectra of the untreated Laspra and corresponding treated samples subjected to SO_2_ action are shown below (Figure 5). In all spectra, the presence of OH stretching from gypsum at 3,546 and 3,405 cm^-1^, as well as the O-H-O bending of the crystallization water at 1,685 and 1,621 cm^-1^ can be evidenced. The sulphate ions can be observed at around 1,141, 1,115, 670 and 602 cm^-1^, respectively, while additional bands corresponding to sulphite ions, at 983 and 944 cm^-1^ can be identified for all commercial products, as well as the untreated Laspra sample. After SO_2_ dry deposition, instead of a decrease of the CO_3_^2-^ asymmetric stretching bands from calcite (1,432 cm^-1^) attributed to calcium carbonate dissolution and formation of gypsum, an important increase in the area of the corresponding bands was evidenced, the most significant one being registered for Laspra coated with Silres (eight times higher).

**Figure 6:**
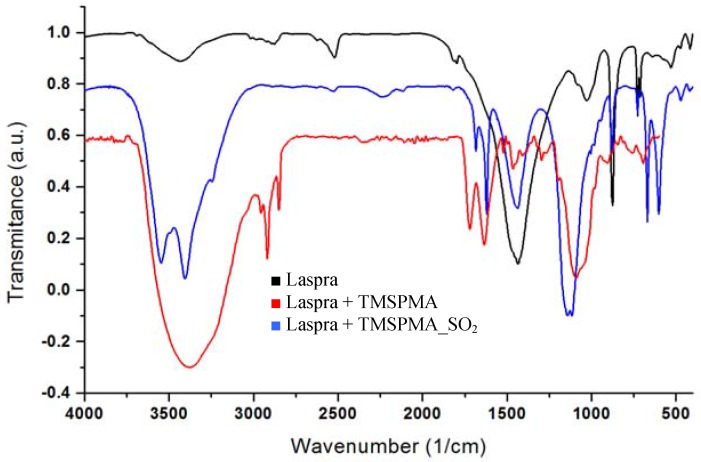
FT-IR spectra of untreated and treated Laspra with TMSPMA compound in comparison with treated Laspra subjected to SO_2_ action.

This result is quite unexpected, at least regarding to TMSPMA action against the untreated Laspra without being subjected to SO_2_ action (Figure 6), where due to the presence of an acidic medium (pH = 5, synthesis conditions for TMSPMA nanocomposite), the CO_3_^2-^ asymmetric stretching from calcite presented a dramatic reduction.

This behavior was attributed to the reaction between calcium carbonate and hydrochloric acid, with the formation of calcium chloride. Upon SO_2_ dry deposition onto Laspra coated with TMSPMA, instead of a further decrease of the CO_3_^2-^ asymmetric stretching from calcite, an important increase of the carbonate region was evidenced (three times higher as compared to the one obtained in the absence of SO_2_).

The carbonate regeneration in case of Laspra can be correlated with the presence of certain sulphur-liberating bacteria in the presence of water. Skoulikidis and Beloyannis reported that gypsum could be converted back to calcite using carbonate anions in aqueous solution [16]. Sulfate-reducing bacteria are able to dissociate gypsum into Ca^2+^ and SO_4_^2-^ ions, and the SO_4_^2-^ ions are then reduced by the bacteria, whereas the Ca^2+^ ions react with carbon dioxide to form new calcite [17] (Equation 3):

6 CaSO_4_ + 4 H_2_O + 6 CO_2 _*→* 6 CaCO_3_ + 4 H_2_S↑ + 2 S + 11 O_2_↑ (3)

**Figure 7:**
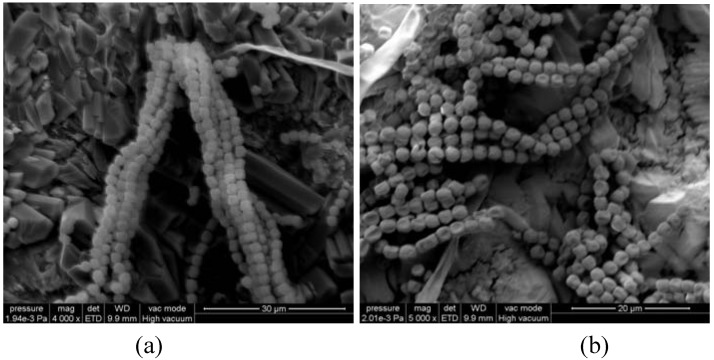
ESEM micrographs of Laspra samples coated with Tegosivin HL 100 at (a) 30 µm and (b) 20 µm after exposure to SO_2_ contaminated atmosphere – evidence of biological growth.

Their presence might explain the abnormal behavior of Laspra samples after the exposure to SO_2_ atmosphere. It is already known that Laspra contains different types of bacteria and microorganisms, responsible for the biological mechanisms that contribute to its deterioration. The higher intensity of the carbonate band for Laspra coated with all compounds and aged can be associated to the enhancement of stone biomineralisation by bacteria (representative images at different resolutions for Laspra samples coated with Tegosivin HL 100, Figure 7), property connected to the water vapor permeability values: the higher the amount of water “entrapped” inside the stone, the higher the amount of carbonate restoration. The smaller increase of the carbonate band in case of Laspra coated with TMSPMA as compared to the worldwide used products can be correlated with the circumstance that the “starting signal” of the carbonate region is very small (around 0.08 on the transmittance scale).

**Figure 8:**
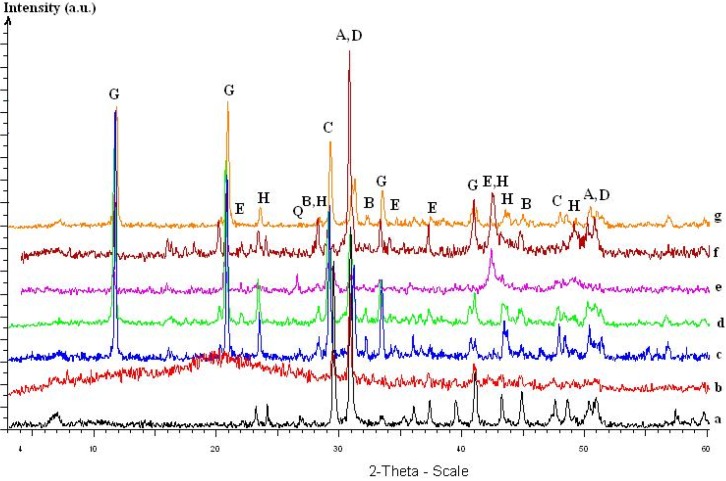
Laspra XRD patterns: (a) untreated. (b) Laspra + TMSPMA without being subjected to SO_2_ action. (c) Laspra + SO_2_. (d) Laspra + Lotexan-N + SO_2_. (e) Laspra + Silres® BS 290 + SO_2_. (f) Laspra + Tegosivin HL 100 + SO_2_. (g) Laspra + TMSPMA + SO_2_.

The X-ray measurements give an overview concerning the qualitative phase identification for Laspra exposed to SO_2_ dry action. As follows, the presence of different phases (dolomite – CaMg(CO_3_)_2_, calcite, ankerite – CaMg_0.32_Fe_0.68_(CO_3_)_2_), sulphite (hannebachite – CaSO_3_·0.5 H_2_O) and sulphate species (gypsum- CaSO_4_·2H_2_O, epsomite - MgSO_4_·7H_2_O, hexahydrite – MgSO_4_·6 H_2_O, kieserite - MgSO_4_·H_2_O, bassanite - CaSO_4_·0.5H_2_O) have been identified. The presence of the commercial products appears to favor the formation of the mineral epsomite (MgSO_4_∙7H_2_O) as one of the reaction products (Figure 8).

G-gypsum, E-epsomite, Q-quartz, H- hannebachite, A- ankerite, D- dolomite, B- bassanite.

Epsomite is frequently able to convert the ambient moisture into liquid water or can lose very easy one molecule of water, with the obtaining of hexahydrite (MgSO_4_·6H_2_O) [18], being responsible for an enhancement of the sulphation process. Therefore, the presence of epsomite only in case of the commercial products may be responsible for the formation of a higher amount of gypsum. In case of Laspra treated with TMSPMA, the crust is made of gypsum and bassanite only. It seems that due to the presence of TMSPMA, SO_4_^2-^ ions balance perfectly with Ca^2+^, leading to the formation of only gypsum as a final product, similar results being obtained on other studies concerning Laspra [19], where MgSO_4_ crystals have identified only after using deionised water.

**Figure 9:**
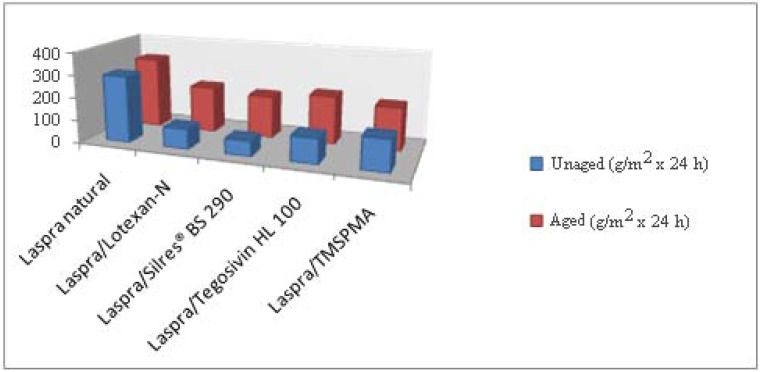
Water vapor permeability – Laspra (L) before and after exposure to SO_2_ atmosphere.

As concerns the data obtained after performing the water vapor permeability experiments (Figure 9), one can state an increase in the water vapor permeability values for all samples. This behavior is mainly due to the migration of the Ca^2+^ ions towards the limestone’s surface leading to the formation of small cavities, to the dissolution of the calcite to form gypsum and to the cracking of the polymeric film. The water vapor permeability coefficients registered before and after the samples exposure to SO_2_ atmosphere indicate that the smallest difference appears for the samples treated with TMSPMA nanocomposite material and the highest increase is shown by the samples treated with Lotexan-N and Silres® BS 290.

**Figure 10:**
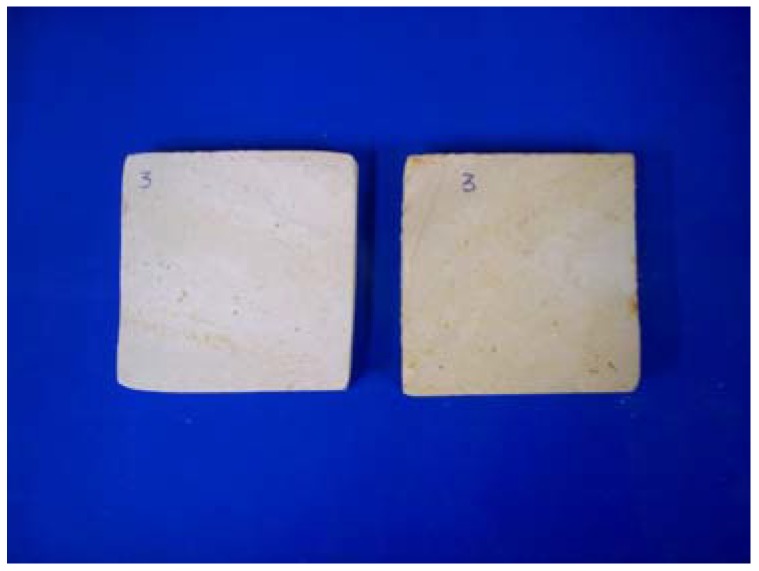
Laspra treated with Tegosivin HL 100 before and after the exposure to SO_2_ action.

Laspra samples are developing a yellowing effect after performing the resistance to SO_2_ action artificially accelerated ageing test, due to the oxidation of the contained iron oxides from Fe^2+^ to Fe^3+^. The iron oxides also act as a catalyst during the sulphation process (Figure 10).

**Figure 11:**
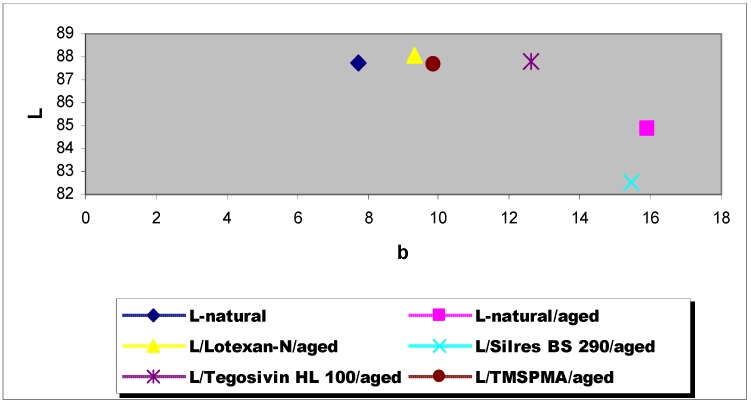
Total color change – Laspra – L* and b* parameters.

The total or global color changes exhibited by Laspra samples after the artificially accelerated ageing under SO_2_ saturated atmosphere are presented in Table 1 and Figure 11.

Table 1 lists the total color change values exhibited by Laspra (natural and treated) after performing the artificially accelerated ageing test. Judging from these data, the most pronounced difference in the total color change occurred for the samples treated with Silres® BS 290, while the samples treated with Lotexan-N, Tegosivin HL 100 and TMSPMA exhibited a small modification.

**Table 1:** Total color change for Laspra – before and after the exposure to SO_2_ atmosphere.

		ΔE*
	ΔE*	after ageing
**Laspra – untreated**		7.07
**Laspra – Lotexan-N**	1.14	1.85
**Laspra – Silres® BS 290**	1.91	7.06
**Laspra – Tegosivin HL 100**	0.75	3.95
**Laspra – TMSPMA**	2.87	1.65

**Figure 12:**
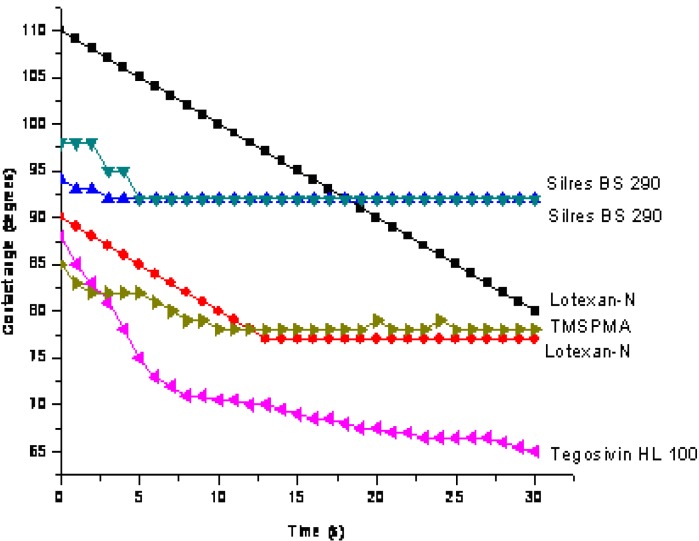
Contact angle values – treated Laspra after exposure to SO_2_ atmosphere.

Figure 12 lists the contact angle values exhibited by Laspra treated samples after the exposure to SO_2_ contaminated atmosphere (initial contact angle values for the unaged treated samples: Lotexan-N – 148°; Silres® BS 290 – 151°; Tegosivin HL 100 – 147°; TMSPMA – 120°).

Tegosivin HL 100 proved to induce either the highest contact angle value, or, on the contrary, the lowest, depending on the degree of degradation of the polymeric film on the particular spot where the water drop was deposited. As for the other siloxane-based water repellents, they show a more constant behavior. For some of the chemical products, the contact angle values registered during the first seconds of the measurement have been much higher then the ones shown by the same product after the 30 seconds of measurement. TMSPMA shows a better behavior in comparison with the commercially available water repellents: the same contact angle values have been registered for a limestone sample, no matter where the water drop was deposited.

## 3. Conclusions

To assess the efficiency of the selected treatments, the water-stone contact angle, water vapor permeability and chromatic values were determined in the limestone specimens before and after treatment. From the comparative assessment of the data obtained in this study, a better protective coating for Laspra treated with the hybrid nanocomposite with silsesquioxane can be pointed out, since it showed a better water vapor permeability value, a reducing of the sulphation degree at the surface and did not alter the color of the stone samples. Due to self-assembling properties, TMSPMA nanocomposite bonds with the limestone and penetrates deeper than the commercial products; therefore, this coating could extend the limestone resistance to SO_2_ action.

For the samples treated with the worldwide used water repellents, the dry deposition of SO_2_ led to the formation of epsomite (MgSO_4_ ∙7 H_2_O) as one of the reaction products, this compound being responsible for the formation of a higher amount of gypsum. Also, the presence of sulphur-liberating bacteria, responsible for the enhancement of stone biomineralisation was evidenced for all samples.

## 4. Experimental Section

### 4.1. Materials

The rock is a highly porous (30% open porosity) white micritic dolomitic limestone, typical for the Spanish region of Asturias, called Laspra, and was used as the main building material of the San Salvador Cathedral of Oviedo. Small inner parts of two IX^th^ century preromanic churches outside Oviedo, namely Santa Maria del Naranco and San Miguel de Lillo were also built from Laspra. These last two churches are included in UNESCO’s Heritage List since 1985. The stone samples were cut in blocks (5 × 5 × 1 cm) and stored in desiccators at 25 °C and 50% relative humidity (RH) for at least 24 h prior to coating application. The coating products were then applied by brushing the stone surfaces with polymer solutions. Following coating application, the stone samples were kept in desiccators at room temperature, at a controlled value of 50% RH. Solvent evaporation was followed gravimetrically until the treated stone specimens reached a steady weight.

The selected commercially available siloxane-based chemical products were Lotexan-N (Keim), a siloxane prepolymer substituted with methoxy, methyl and alkyl groups, dissolved in a mixture of aromatic/aliphatic hydrocarbon solvents, Silres BS 290 (Wacker Silicones), a mixture of silanes and siloxanes, applied using white spirit as solvent, and Tegosivin HL 100 (Goldschmidt), a low molecular weight modified polysiloxane resin (methylethoxypolysiloxane) whose application requires the dissolution in white spirit. The hybrid nanocomposite with silsesquioxane units (TMSPMA) was obtained combining the sol-gel technique and the radical polymerization of an alkoxysilane sol-gel precursor, namely 3-(trimethoxysilyl)propyl methacrylate, in the presence of a primary amine surfactant [7,8]. The coating products were applied onto stone surfaces with polymer solutions of 11% w/v for the commercial products and 7% w/v for the compound TMSPMA.

### 4.2. Measurements

The resistance of the stone samples against SO_2_ action was determined by placing the stone samples in a acid resistant container during 21 days. For a container of 50 liters:

solution: 500 ± 10 mL H_2_SO_3_ diluted in 150 ± 10 mL H_2_O
m_0_ – dry sample weight before the experiment (g)
m_1_ – dry sample weight after the experiment (g)
Δm – weight variation (%) (Δm = [(m_0_ – m_1_) / m_0_] × 100).

The stone samples were submerged in water at 20 ± 5º C for 24 hours prior to the experiment. The samples were then introduced in a container having, on its bottom, the acidic solution. The stone samples were placed at least 100 mm higher than the acidic solution level. The container was closed and sealed in order to maintain the SO_2_ saturated atmosphere during the 21 days of experiment. After 21 days, the stone samples were washed and dried until reaching constant weight.

The thickness of the gypsum crust was determined by the following expression (Equation 4):

δp = Wp/A∙ρ_p_ (4)

where:

δp = crust thickness (cm),
Wp = weight of the product, gypsum (g)
ρ_p_ = density (g cm^−^^3^) of gypsum, 2.32
A = surface area of the sample, cm^2^.

For water vapor permeability measurements, the sample blocks were fixed on the top of identical cylindrical polyvinyl chloride (PVC) containers that were partially filled with distilled water. The containers were afterwards placed in a desiccator, kept at a value of 25% RH and at constant temperature (20 °C). The containers were weighted every 24 h, for 7 days. It was assumed that the vapor flow through the stone had reached a constant value when the difference between two consecutive daily (24 h) weight variations was less than 5%. The permeability coefficients (k_v_) were calculated according to the following formula (Equation 5):

(5) $$k_{v}=-\frac{\Delta M/S}{t\;(g/m²\cdot 24h)}$$

where - (ΔM/S) = - (M_t_ - M_o_) / S (g/m^2^), M_0_ - the initial container mass at t = 0, (g), M_t_ - the container mass at Δt = 24 h; for t = 0, M_t_ = M_o_, (g); S = 0.00159 m^2^ (standard value).

The optical characteristics were evaluated through color alteration measurements taken on homogeneous spot areas using a portable MiniScan XE Plus (HunterLab Associates Inc., USA) reflectance spectrophotometer and were determined by the use of L*, a* and b* coordinates of the CIE 1976 scale. Color measurements are expressed using CIE L* a* b* and CIE L* C* h systems, where L* represents the variable lightness, which can vary from 0 (black) to 100 (white), a* and b* are the chromatic coordinates, *i.e.*, + a is red, – a is green, + b is yellow and – b is blue. The attributes of chroma are C* – saturation or color purity, and hue h – color wheel. The global color variation (ΔE) was evaluated using the formula ΔE* = (ΔL*² + Δa*² + Δb*²)^1/2^.

The thickness of the coated layers on the limestone surface was determined through ESEM micrographs (cross-sectioned samples), as follows: 16.2 µm (Lotexan-N), 13.9 µm (Silres® BS 290), 9.6 µm (Tegosivin HL 100) and 3.6 µm (TMSPMA).

### 4.3. Characterization

FTIR spectra were performed on a Bruker Vertex 70 instrument, in the 400–4,000 cm^−1^ region, 64 scans, at room temperature, using the KBr pellet technique and the Opus 5 FTIR Software. The spectra were recorded at a resolution of 2 cm^−1^ and a incidence angle of 45°. The signal-to-noise was improved by co-adding 128 scans/spectrum. Peak height measurements were performed with the spectral analysis software (Opus 5). The areas for CO_3_^2-^ asymmetric stretching peak after and before reaction, calculated by Gaussian fitting, are the following: natural Laspra – 9.3; Laspra/TMSPMA – 3.8; Laspra/TMSPMA + SO_2_ – 1.2. The ESEM micrographs were obtained with a Quanta 200 scanning probe microscope, the specimens being fixed with adhesive past on Al conducting supports of cylindrical shape and then sputter-coated with gold. X-ray patterns were recorded with a D8 Advance Bruker AXS diffractometer and the diffractograms were studied with an EVA soft (from Diffrac Plus evaluation package). X-rays were generated using a CuKα source with an emission current of 40 mA and a voltage of 36 kV.
